# Geographical and environmental approaches to urban malaria in Antananarivo (Madagascar)

**DOI:** 10.1186/1471-2334-10-173

**Published:** 2010-06-16

**Authors:** Fanjasoa Rakotomanana, Jocelyn Ratovonjato, Rindra V Randremanana, Laurence Randrianasolo, Rogelin Raherinjafy, Jean-Paul Rudant, Vincent Richard

**Affiliations:** 1 Unité Epidémiologie, Institut Pasteur, BP 1274, Antananarivo, Madagascar; 2 Unité d'Entomologie médicale, Institut Pasteur, BP 1274, Antananarivo, Madagascar; 3 Unité de Recherche sur le Paludisme, Institut Pasteur, BP1274, Antananarivo, Madagascar; 4 Institut Francilien de Géosciences, Université Paris-Est, Marne La Vallée, France

## Abstract

**Background:**

Previous studies, conducted in the urban of Antananarivo, showed low rate of confirmed malaria cases. We used a geographical and environmental approach to investigate the contribution of environmental factors to urban malaria in Antananarivo.

**Methods:**

Remote sensing data were used to locate rice fields, which were considered to be the principal mosquito breeding sites. We carried out supervised classification by the maximum likelihood method. Entomological study allowed vector species determination from collected larval and adult mosquitoes. Mosquito infectivity was studied, to assess the risk of transmission, and the type of mosquito breeding site was determined. Epidemiological data were collected from November 2006 to December 2007, from public health centres, to determine malaria incidence. Polymerase chain reaction was carried out on dried blood spots from patients, to detect cases of malaria. Rapid diagnostic tests were used to confirm malaria cases among febrile school children in a school survey.

A geographical information system was constructed for data integration. Altitude, temperature, rainfall, population density and rice field surface area were analysed and the effects of these factors on the occurrence of confirmed malaria cases were studied.

**Results:**

Polymerase chain reaction confirmed malaria in 5.1% of the presumed cases. Entomological studies showed *An. arabiensis *as potential vector. Rice fields remained to be the principal breeding sites. Travel report was considered as related to the occurrence of *P. falciparum *malaria cases.

**Conclusion:**

Geographical and environmental factors did not show direct relationship with malaria incidence but they seem ensuring suitability of vector development. Absence of relationship may be due to a lack of statistical power. Despite the presence of *An. arabiensis*, scarce parasitic reservoir and rapid access to health care do not constitute optimal conditions to a threatening malaria transmission. However, imported malaria case is suggestive to sustain the pocket transmission in Antananarivo.

## Background

Urbanisation universally accompanies demographic growth and is currently accelerating in Sub-Saharan Africa. The urban population was estimated at 48% of the world population in 2003 and 50% in 2007 [1,2]. All countries are faced with the reality that, between now and 2050, three billion people are likely to settle in urban areas, principally cities. This rapid growth of urban populations is due to an exodus from rural areas and the progressive urbanisation of the rural areas around existing cities. It mostly concerns developing countries, in which the growth rate of the urban population is higher than that of the general population. The growth rate of the general population has been estimated at 3% in the South, whereas the urban population is growing at a rate of 6% or 9% in some African countries [3]. The urban areas of developing countries are experiencing disproportionate growth in the numbers of humans and expansion, with all the associated planning problems associated with uncontrolled growth.

Urbanisation would influence the epidemiological characteristics of diseases. For example, urban malaria depends on the suitability of environment to mosquito development: availability and quality of breeding sites. Several studies in Africa have investigated the factors contributing to the maintenance of anopheline mosquito populations in urban areas. In Dakar, Senegal, market-garden wells constituted major larval sites for *An. arabiensis *[4]. Trape and Zoulani (1987) found ditches, gutters and tyre tracks to be important sites for anopheline larvae in Brazzaville, Congo [5]. In a newly urbanised area of Western Kenya, Khaemba *et al*. found that *An. gambiae *larvae were more likely to be found in human-made temporary pools of water, such as tyre tracks and ditches [6]. A study carried out in two urban areas of Kenya showed that 65% (Kisumu) and 93% (Malindi) of the aquatic mosquito habitats were created by humans and that 39% and 65% of these habitats harboured anopheline larvae. Studies have used remote sensing to identify mosquito breeding sites. High-resolution remote sensing data can be used to generate a digitized grid for the random sampling of breeding sites. Integration of the results into a GIS makes it possible to study the relationship between mosquito breeding site availability, vector abundance and land use-land cover change [7-9]. Carter *et al. *(2000) investigated the relationship between malaria risk and vector breeding sites [10]. Carter *et al. *(1998) and Thomson *et al. *(1996, 1997) described the relationship between malaria risk and environmental indices obtained by remote sensing [11]. Omumbo *et al. *(1998) used a GIS to quantify the relationship between the occurrence of anopheline mosquitoes and environmental variables [12].

The main epidemiological facies (features) of malaria in Africa are found in Madagascar. The equatorial facies on the East Coast is characterised by a transmission all year long. In the tropical facies on the West Coast, transmission is seasonal (seven months at least). In both areas, the transmission is stable. In the Southern facies, the transmission is seasonal (two to four months per year). In the plateau facies, higher than 1,000 m above the sea level, the transmission is unstable. The population in the central highlands is vulnerable to epidemics. Until 1895, two malaria epidemics occurred in the highland areas. In 1949, an eradication program was launched based on Dichloro-Diphenyl-Trichloroethane (DDT) house-spraying and chloroquine prophylaxis in children. The malaria control activities declined gradually in the 1970s. *An. funestus *re-invaded the Central Highlands, causing the last malaria epidemic at the end of the 1980s. Antananarivo is situated in the central highlands of Madagascar and presents a plateau facies [3,13]. Several presumed cases have been registered at the urban health centres, but most of them were not confirmed by parasitological examination. Rice fields are a favourable environment for mosquito larvae in Antananarivo and are the breeding site of anopheles mosquito [14-17].

Two cross-sectional surveys conducted in the urban of Antananarivo, showed the confirmation of some cases of malaria: 1.9% in February 2003 (wet season) and 1.5% in July 2003 (dry season). Of the 15 cases identified in February, only two, both caused by *P. falciparum*, were considered likely to be indigenous (0.26%). Three cases in July were considered to be of local origin (0.4%). The study showed the prevalence of cases to be very low, although some cases of indigenous malaria were also observed [18].

Previous studies have mostly focused on the prevalence of malaria among the population attending health centres. Our main motivation for studying the relationship between geographical and environmental factors and the incidence of confirmed malaria cases in an urban setting was to understand the impact of these factors on the pattern of malaria cases. In this study, we aimed to update maps of rice field location, using recent remote sensing data, and to investigate the available breeding mosquito sites. Then we investigated the contribution of environmental factors to the incidence of urban malaria in Antananarivo.

## **Methods**

### Study sites

The study was conducted in Antananarivo, the capital of Madagascar. This city extends over 80 sq km and its population was estimated at 1,697,000 inhabitants in 2007 [19]. The urban area of Antananarivo is located on a vast alluvial plain, extending from 18°48'S and 47°24'E in the North West, to 19°00'S and 47°42'E in the South East. The urban area consists of administrative, commercial, industrial and residential areas, with patches of agricultural land, most of which are used to grow rice. Antananarivo is crossed by the river Ikopa and canals (irrigation, drainage). Farmers make use of rainwater-fed irrigation systems, which are carefully managed, both communally and individually, to ensure that the rice fields receive adequate water supplies. The rest of the plain contains rice fields, which display dynamic changes during the growing season. Uncultivated rice fields are left flooded, or are allowed to become overgrown with weeds or reeds.

This area has a tropical, high-altitude climate, with a cold, dry season from May to October and a hot and wet season from November to April. The mean annual temperature is 18°C, with a maximum in November (26°C) and a minimum in July (10°C). Annual rainfall ranges from 1,000 mm to 1,600 mm per year.

**Figure 1:**
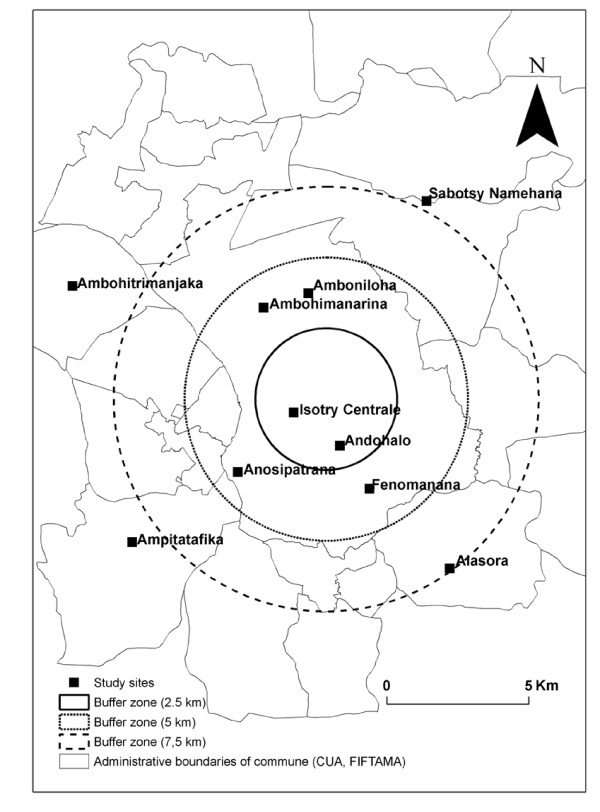
Location of study sites related to distance from the city centre (epidemiological and entomological sites).

The study area included the dense urban core of Antananarivo, consisting of six districts, known as the urban municipality, and the surrounding suburbs. The suburbs of Antananarivo contain 28 municipalities in the public office of inter municipal cooperation. The suburbs and the six districts constitute the "Great Antananarivo". The urban municipality has 16 public health centres. Five were selected to participate in the study: one in the centre and three located about 5 kilometres from the city centre. Each of the considered suburbs has a health centre, and four centres were selected for study (10-15 kilometres from the city centre; Figure 1). Health centres were selected on the basis of their geographical location and their inclusion in the public health system, making them accessible to the general population (health care costs lower than those charged by private health centres). One of the centres is run by a religious group. It participated in previous study that one observed positive malaria case without stay outside Antananarivo.

### Geographical data

The geographical data collected included an Enhanced Thematic Mapper plus (ETM+) image from Landsat 7, with bands 1 to 7. The ETM+ image from Landsat 7, provided by USAID, was acquired in May 2000, at the beginning of the dry season. It is a georeferenced image, with a pre-processing level of 1G and a resolution of 30 m. Two Advanced Synthetic Aperture Image Mode Precision (ASA_IMP_1P) images from Envisat, Digital Elevation Model (DEM) topographic maps and data from the development office of Antananarivo were used to update data concerning the location and extent of rice fields. The images from Envisat were acquired in January 2004, early in the development of the rice crop, and in July 2004, when rice was mature or had already been harvested. These images were multi-look, C-Band, digital images, with vertical/vertical (VV) polarisation and a resolution of 25 m. These images were acquired from the CAT1-2320 Project of the European Space Agency. The DEM was provided by the European Space Agency in 2004, as part of the Epidemio Project, and was obtained from Advanced Synthetic Aperture Radar data from Envisat with a resolution of 50 m. Nine topographic maps from the national institute of geography and hydrography were used for fieldwork, to complete the visual interpretation of images for the location of training and test sites. The scale of these maps was between 1:10,000 and 1:50,000. The geographical coordinates of these sites were determined with a handheld global positioning system (GPS). The development office of Antananarivo provided population data, hydrological networks and data concerning the administrative boundaries of the "fokontany" (neighbourhood, the smallest administrative district).

### Climatic data

Climatic data were available from the National Meteorological Service and included minimum, maximum and mean temperatures and rainfall data. These data present normal monthly temperatures and rainfall for a period of 30 years (from 1971 to 2000) and constitute the reference values for climatic data. Average climate predictions, by 10-day period, for 2006 to 2007, were obtained from the African Centre of Meteorological Application for Development.

### Location of rice fields

The maximum likelihood method was used for classification purposes, with the Landsat 7 image. This method was adopted because the training sites were well chosen and corresponded to highly homogeneous sites. Classification accuracy was estimated by calculating the Kappa coefficient and the confusion matrix for the training class pixel. Radar images taken on two different dates were used to complete the results. The backscatter coefficient was calculated as follows: σ_0 _= 10 × log 10 (pixel value). The image from the wet season (January) was compared with the image from the beginning of the dry season (July), to assess the differences between the two periods. Thresholds were identified for the assessment of changes, by matching the values of backscatter coefficients with the type of landscape. Changes were identified on the basis of the combination of colours from the two images. The classification generated with the optical data was improved by results from radar images, which removed ambiguities relating to whether certain areas were covered by rice fields or other types of vegetation.

Field surveys were carried out on two occasions, for environmental interpretation: i) Training and test sites were identified. The co-ordinates of the rice fields were recorded with a handheld global positioning system (GPS). ii) Checking, on the ground, that areas identified as rice fields really were rice fields.

### Entomological study

Entomological studies were conducted at six sites (two in the centre, two in neighbouring districts and two in suburb municipality). At each study site, we surveyed temporary, permanent, and semi-permanent breeding sites and mosquito larval populations at the beginning, middle and end of the wet season (November 2006, January and March 2007, respectively). Samples of mosquito larvae were collected from aquatic habitats at each site. The larvae were collected in a white dish [20] if the breeding site was shallow, or with a small nylon gauze net mounted on a circular frame (15 cm in diameter) and attached to a wooden handle (1 m) if the breeding site was deeper. The use of a gauze net ensured that we did not lose the small larvae, and this approach was particularly useful for rice fields [21]. The larvae were transferred to 250 ml plastic bottles containing water from the breeding site, for transfer to the laboratory at the Institute Pasteur of Madagascar, where they were housed in insectariums until emergence.

Seven houses located within 200 m of the nearest large breeding site were randomly selected and sampled for adult mosquitoes by pyrethrum spray catches (WHO 1975). The collections were conducted between 6.00 and 10.00 am on three separate days. Adult anophelines from pyrethrum spray catches and larval surveys were identified morphologically to species [22] and preserved dry in vials containing a desiccant (silica gel). Sibiling species of *Anopheles gambiae s.l. *were identified to species using rDNA-PCR method and the protocol described by Scott *et al*. [23]. DNA was extracted from the legs of mosquitoes as described by Collins et *al *[24]. The heads and thoraces of all female anophelines were screened by Enzyme-linked immunosorbent assays (ELISA) for Plasmodium falciparum circumsporozoïte proteins [25].

### Epidemiological study

The epidemiological survey began in November 2006 and continued until December 2007. Clinical examinations were carried out by clinicians, on the basis of the presumed malaria case definition of the National Malaria Control Programme: temperature ≥37°5C in the absence of symptoms associated with other diseases. Thick blood smears were collected on blotting paper discs the size pieces of confetti, from patients with presumed malaria who had given informed consent. Additional information was collected by questionnaire: patient identification, social and economic status (type of dwelling, means of water supply, use of mosquito nets etc.), travel details (number of journeys, date and place) and history of malaria (number of clinical episodes, treatment received, source of treatment) in the previous three months. The travel report provided information on periods away from Antananarivo in the three months before the consultation. Blood samples were collected and questionnaires were completed each month by a local consultant at each site. PCR identified genuine cases of malaria from the blood samples supplied by patients. Real-time PCR based on the Mongold technique was performed in the Rotor-Gene 3000 machine, using the intercalating agent SYBR Green I as the fluorescent marker [26].

A school survey was carried out in March 2007 at the sites studied (centre, immediate vicinity and suburbs). We investigated one school in each area, selecting those nearest the health centres. We examined 200 school children between the ages of six and ten years at each school, with their parents' consent. Rapid diagnostic tests were used to test for malaria in febrile school children (temperature ≥37.5°C). Children testing positive were treated according to national guidelines.

This study was approved by the national ethics committee (No. 005-SANPF, delivered by the ministry on January, 8^th ^2007).

### Determination of risk factors

Antananarivo is a city geomorphologicaly contrasted; one part the hilly zones, often with steep slope liable to erosion and the other part the low zone liable to flooding. The lower area liable to flooding is defined as an area with an altitude not exceeding 1,250 m. This value corresponds approximately to the alert status of the disaster contingency plan in case of flood [27]. Part of these areas consists of rice fields or wetlands. These characteristics led us to consider the altitude, population density and rice fields as factors influencing malaria in our input data. The population density was calculated in relation to habitable areas because of large tracts of farmland.

A geographical information system was constructed for the integration and manipulation of geographical data (altitude, rice field surface area), field data (entomological and epidemiological) and other spatial referenced data (population density, health centres). Kruskal-Wallis tests were used to assess the effects of these factors on the occurrence of confirmed malaria cases. Correlations between the factors studied and malaria cases (with and without stay outside Antananarivo) were studied at 15-day intervals over the year-long study period. Risk analysis was carried out at two levels: at the level of the individual, based on social and economic status, and at district level, based on the presence of confirmed malaria cases in the neighbourhood, with or without reported travel. Chi square tests were used to assess the significance of differences between positive and negative cases of malaria, as a function of the factor considered. McNemar matched pairs tests, taking the neighbourhood as its own control, were performed to assess risk at the level of the neighbourhood.

Monthly averages were calculated for temperature. Total were calculated for monthly rainfall. Temperature and rainfall during the study period were compared with normal values, in Spearman's rank correlation analysis. We then analysed the correlations between temperature and confirmed malaria cases and between rainfall and confirmed malaria cases. Climate data were studied separately from the other factors because of difference of spatial scale. Data on altitude, population density and rice fields are available by neighbourhood while climate data concern the entire study area.

## Results

### Location of rice fields

Fieldwork was carried out to observe the various stages of cultivation in rice fields (fallow, rice nurseries, flooded rice fields, ploughed rice fields), the type of crop (differentiation between rice plantations and other crops) and the type of vegetation (grass, trees, shrubs). Supervised classification of the Landsat 7 image by the maximum likelihood method resulted in an overestimation of the area under rice fields, due to the alternation with other vegetation. This method confused rice fields, marshes and trees (young eucalyptus trees). The map obtained was found to be 87% accurate.

**Figure 2:**
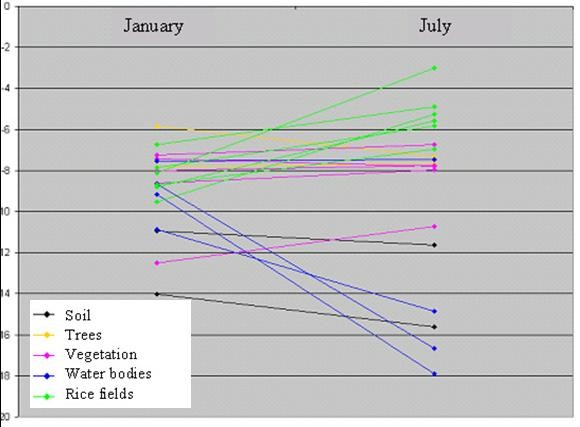
**Evolution of backscatter coefficient between ASAR_IMP_1P of January and July 2004. **The backscatter coefficient of rice field increased, open water decreased and urban backscatter coefficient remained relatively unchanged.

**Figure 3:**
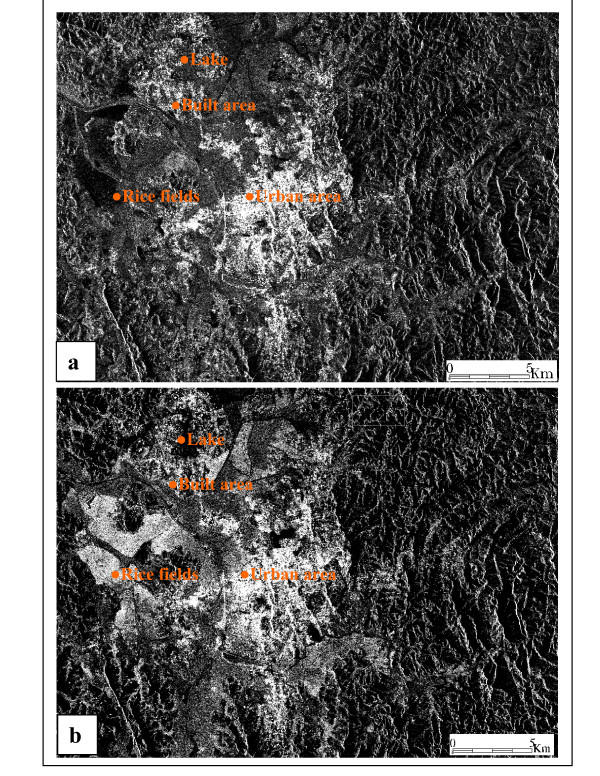
**ASAR_IMP_1P images illustrating changes between January and July 2004. **Difference between images acquired in January and July 2004.

For radar images, rice fields and open water were the landscapes displaying the most significant change in backscatter coefficient between the two recording periods. The backscatter coefficient of rice fields increased, whereas that of open water decreased between January and July (Figure 2). In January, the fields were flooded and the radar image was dark. The backscatter coefficient increased progressively with rice growth. Mature rice, young seedlings and fields ploughed for a second cycle of cultivation gave a more intense signal in July. No change in radar signal between these two months was observed for urban and built-up areas (Figures 3).

The rice fields identified from the two radar images were more restricted in area than those identified by optical image classification. As the rice fields were mostly located at the bottom of the valley, all areas located higher up the mountains were removed with the DEM.

### Entomological study results

*An. arabiensis *was identified as a potential vector. It was collected from three zones of the urban area: the centre, neighbouring districts and the periphery. The identification of adult mosquitoes reared from larvae in the laboratory revealed an absence of *An. funestus*, the mosquito responsible for the last epidemic of malaria. Rice fields remain the principal breeding sites for mosquitoes at the start and in the middle of the wet season, accounting for 46% and 56%, respectively, of the identified breeding sites. The next most frequent type of breeding site was ground pools created by the removal of earth for brick production. *An. arabiensis *was the predominant species among the mosquitoes caught in houses.

At the start of the wet season, mosquito larvae were found in 26.5% of the breeding sites prospected, with 62.9% of the larvae identified belonging to the *Culex quinquefasciatus *species, and 37.1% belonging to *An. arabiensis*. During the wet season, mosquito larvae were found in 34.1% of the prospected breeding sites. These larvae belonged to the following species: *An. arabiensis *(42.4%), *Culex quinquefasciatus *(36.4%), *Culex poicilipes *(15.7%), *Culex antennatus *(3.8%), *An. squamosus *(0.2%). At the end of the wet season, only 13.8% of the breeding sites contained mosquito larvae. The predominant species at this time point were *Culex quinquefasciatus *(51.9%), followed by *Culex antennatus *21.5%) and *An. arabiensis *(12.5%).

No infectious female mosquitoes were found. The results of the entomological study are reported elsewhere (Ratovonjato *et al. *2009, *in preparation*).

### Epidemiological results

Over a period of 14 months, we recruited 1,108 subjects. These patients were aged between three months and 87 years and had a mean age of 21 years. The sex ratio was 0.8 (male/female).

The healthcare centres serve 343,507 people in total, 75,859 (22.1%) of whom consulted. The annual incidence of malaria was 14 cases/100,000 inhabitants. Cases of presumed malaria accounted for 1.4% (1,108 patients) of consultations, and 5.1% of these presumed cases were confirmed positive by PCR. The proportion of presumed malaria cases subsequently confirmed by our analysis is shown in table 1. Four *Plasmodium *species were detected, *P. Falciparum *was found as the most prevalent specie including mixed infections 73.7% (n = 42/57).

**Table 1:** Number of confirmed malaria cases related to presumed malaria cases and population in the neighbourhood (November 2006 - December 2007)

Observation	Percent (n= number)
Population	343507
Consultations/population	22.1 (n= 75 859)
Presumed malaria cases/consultations	1,4 (n = 1108)
Confirmed malaria cases/Presumed malaria cases	5,1 (n = 57)
*Plasmodium falciparum*/consultations	3.8 (n = 42)
*Plasmodium vivax*/consultations	0.8 (n = 9)
*Plasmodium malariae*/consultations	0.4 (n = 5)
*Plasmodium ovale/*consultation	0.1 (n = 1)

Of the confirmed malaria cases 52.6% (n = 30/57) were declared "indigenous cases", 45.6% (n = 26/57) had stayed outside the urban area of Antananarivo. No information about travel was available for one confirmed case (1/57). Considering "indigenous cases", *P. falciparum *remained the most prevalent species (50.0%), followed by *P. vivax *(23.3%), *P. malariae *(10.0%) and *P. ovale *(3.3%). Mixed infections were also found: *P. falciparum/P. malariae *(10.0%) and *P. falciparum/P. vivax *(3.3%).

In the school survey, we examined 83.2% (1,664/2,000) of the schoolchildren, parental consent having been obtained for the analysis of these children. Only 74 (4.5%) of these children were febrile and only one of these febrile children tested positive for malaria by RDT. This case was probably contracted during a documented week away from Antananarivo.

### Risk analysis

**Figure 4:**
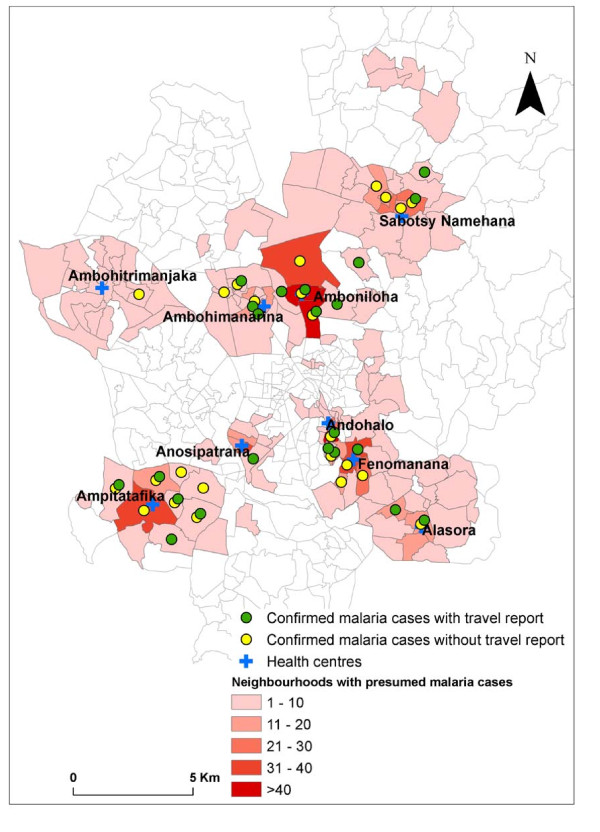
Neighbourhood distribution of malaria cases; each symbol represents one or two confirmed malaria cases.

Individual risk analysis identified no significant risk factors (Table 2). For *P. falciparum *infection, a stay outside Antananarivo was identified as a risk factor for the occurrence of positive malaria cases (p < 10^-7^). Confirmed *P. falciparum *malaria was significantly more frequent in patients who had reported stay outside Antananarivo than in "indigenous cases". The distribution of confirmed malaria cases as a function of neighbourhood is shown in figure 4. Several neighbourhoods had both "indigenous" malaria cases and cases reporting travel (n = 11). Some neighbourhoods had "indigenous" cases but no cases reporting travel (n = 10). Conversely, others had cases reporting travel but no "indigenous" cases (n = 11). Finally, some districts had no confirmed malaria cases (n = 152). The analysis of the relationship between "indigenous" cases and "imported or introduced" cases showed no statistically significant relationship between "indigenous" malaria cases and cases reporting travel.

**Table 2:** Analysis of individual risk factors of positive malaria cases (*P. falciparum*) with no travel report

Variables	Positive cases N (%)	Negative cases N (%)	P value
Sexes			NS*
*Female*	11 (57.9)	589 (56.1)	
*Male*	8 (42.1)	461 (43.9)	

Age (mean)	23.1	21.5	NS

Socio economic states			NS
1 *Wall*: mud, wood or iron	5 (26.3)	389 (37.0)	
*Water supply*: river, well			
2 *Wall*: brick, concrete	7 (36.3)	294 (28.0)	
*Water supply*: public tape water, well			
3 *Wall*: brick, concrete	7 (36.8)	368 (35.0)	
*Water supply*: inside tape water			

Proximity of agricultural area	12 (63.2)	532 (50.6)	NS

Bed net use	4 (21.1)	294 (28.2)	NS

*: Not significant

Medical care began early, with patients consulting a health centre within three days of the onset of clinical symptoms, on average.

**Figure 5:**
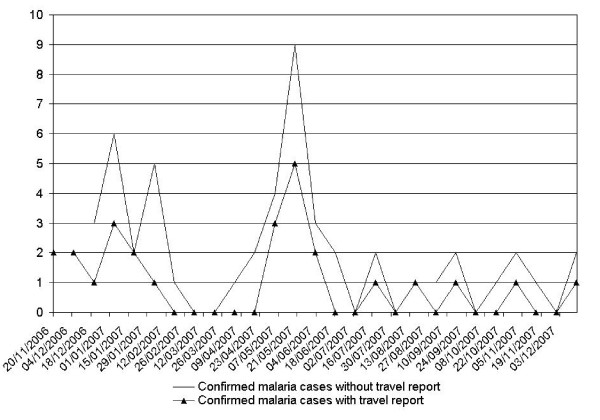
Distribution of confirmed malaria cases at fifteen day intervals.

Analyses of confirmed malaria cases at 15-day intervals showed relationship for the occurrence of "indigenous" cases after that of cases reporting travel outside Antananarivo (Figure 5). Spearman's rank test showed correlation between indigenous confirmed malaria cases and those with stays outside Antananarivo, r = 0.37 and p < 0.05. Checking address of patients showed no relation to each other. An increase of malaria cases was observed in April (wet season). The 6 districts and 28 suburban towns that make up the urban area of Antananarivo contain 454 neighbourhoods. The presumed malaria cases observed in the health centres participating in the study come from 153 neighbourhoods (34%). The study area was located at an altitude of 1,243 m to 1,596 m. Altitude of the 153 neighbourhoods varies from 1,247 m to 1,381 m ; of which altitude equal or less than 1,250 m are 21% (32/153). Areas of altitude less than 1,250 m were vulnerable to flooding during the strong rainfall period. The urban areas at these lower altitudes were essentially slums, characterised by low incomes. Population density of considered neighbourhoods varies from 916 inhabitants per sq km to 111,975 inhabitants per sq km and rice fields' area varies from 0 to 3.7 sq km. Table 3 showed the characteristics of these considered factors. We found that the presence or absence of confirmed malaria cases was not affected by the altitude of the neighbourhood, population density or the area of the rice fields.

**Table 3:** Global neighbourhood characteristics based on factors considered

Total of neighbourhoods	454
Neighbourhood of presumed malaria case (%)	34 (n = 153/454)

Altitude average (metre)	
- Minimum	1247
- Maximum	1381

Density of population (inhabitants/sqkm)	
- Minimum	916
- Maximum	11975

Rice fields, including wetland (sqkm)	
- Minimum	0
- Maximum	3.7

**Figure 6:**
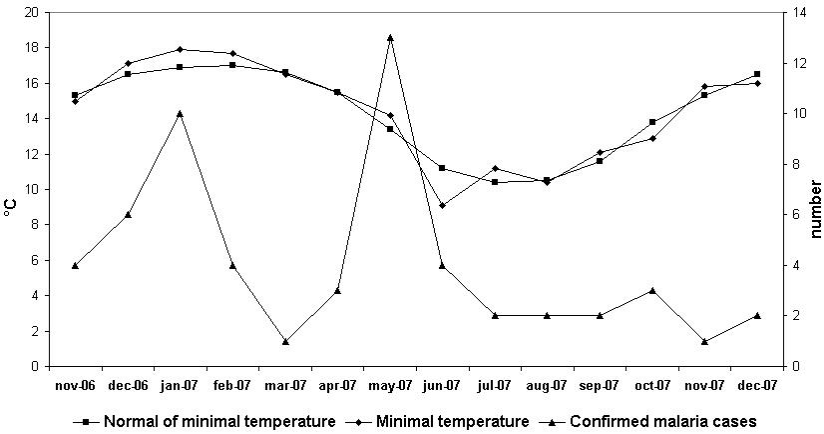
Minimum temperature and confirmed malaria cases during the study period.

**Figure 7:**
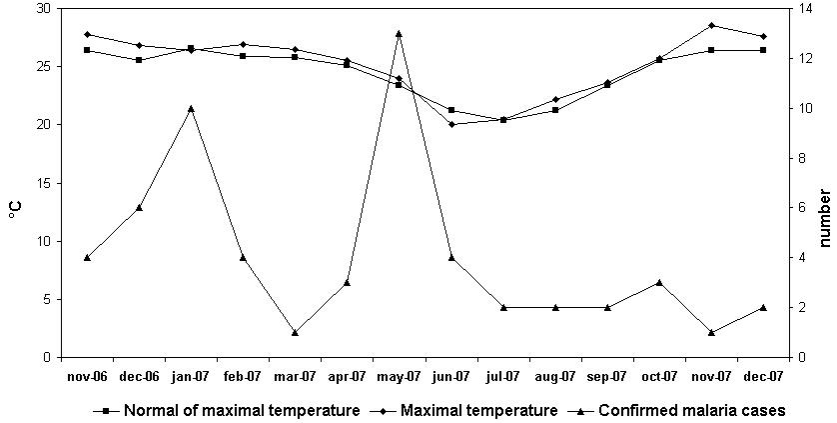
Maximum temperature and confirmed malaria cases during the study period.

**Figure 8:**
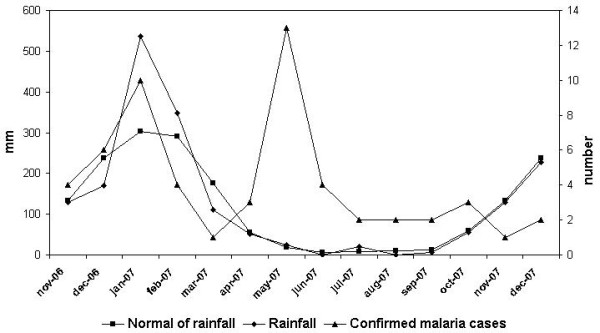
Rainfall and confirmed malaria cases during the study period.

The period with the highest temperatures was not associated with confirmed malaria case, but rainfall was followed by confirmed malaria case. Temperature was not significantly related to the number of confirmed malaria cases (Figure 6, Figure 7). However, we obtained typical curves for the incidence of malaria, showing infections associated with the wet season, with a peak at the start of the wet season in November and another peak towards the end of the wet season (after the floods) (Figure 8). Spearman's rank correlation analysis showed that the maximum and minimum temperatures and rainfall recorded during the study period were strongly correlated with normal maximum temperatures (p < 10^-4^; r = 0.88), minimum temperatures (p < 10^-6^; r = 0.96) and rainfall (p < 10^-6^; r = 0.96).

## Discussion

Antananarivo consists of mixture of urban and rural areas, in which mosquito breeding sites are available. Rice cultivation is associated with practices resulting in the maintenance of mosquito habitats. This study demonstrates the benefits of combining optical and radar images to obtain more accurate information on breeding sites availability. However, the results obtained were more suitable for assessing wetland than for the fine mapping of rice fields. The vegetation colonising marshes may also be mapped. We considered rice fields and other wetlands to have similar environmental characteristics in terms of mosquito breeding sites.

The study showed that *An. arabiensis *was identified as a potential vector. Absence of *An. funestus *and infected vector was observed. Direct relationship was not observed between geographical and environmental factors and confirmed malaria cases but their influence on vector development is well known. Altitude of neighbourhoods ranges from 1,243 m without exceeding 1,500 m. Above 1,500 m of altitude, one know that low temperature blocks the intrinsic cycle of parasite in mosquito. Altitude was important because low-lying zones (below 1,250 m) were liable to flooding and were thus suitable sites for mosquito breeding after strong rainfall (ground pools). The effect of rainfall was observed with cases recrudescence during wet season. We found that rice fields were the most important breeding sites for *An. Arabiensis *even if association with confirmed malaria case was not observed. It may influence the development of mosquito vector. Similarly, the density of the population does not show a direct relationship with the incidence of malaria while the high population density may result by pollution and reducing availability of breeding sites. The impact of population density can be assessed indirectly, by measuring land use-land cover change using remote sensing.

These findings give evidence of suitability to vector development. Association with imported malaria cases is likely to ensure the presence and maintaining of local transmission. Analyses of confirmed malaria cases at 15-day intervals showed significant correlation between "indigenous" cases and cases reporting travel outside Antananarivo. This is suggestive the effect of a constant malaria importation from lowlands and nearby rural areas that is able to sustain low-level transmission in Antananarivo. On the other hand, neighbourhoods with or without imported malaria cases are close enough. Rapid access to health care may break down a probable transmission. Medical care began early, with patients consulting a health centre within three days of the onset of clinical symptoms, on average. That results by low rate of malaria incidence in the urban of Antananarivo. We obtained low incidence of malaria (5.1%) and confirmed the occurrence of malaria cases unrelated to travel. The low incidence precluded the assessment of transmission, although our results suggest intra-urban variation of the transmission pattern.

In tropical countries, such as Madagascar, it is difficult to acquire images with optical sensors, due to permanent cloud-cover during the wet season, which may affect the quality of the images obtained. Radar remote sensing has the advantage of being able to penetrate clouds, making data acquisition possible at any time, day or night, in all weather conditions. We realise that our results included wetlands as well as rice fields. Kaya *et al*. reported similar problems of confusion between wetlands and mangrove forests. These two classes had similar target geometry and moisture conditions, making them difficult to separate [28]. One of the difficulties in risk factor studies is the problem of locating breeding sites, due to the limited resolution of satellite images. It is difficult to use such images to detect the small pools constituting urban breeding sites. The lack of standardised data collection is an additional problem [29]. We therefore did not take into account the validity of the times at which our data, such as satellite images and population density, were obtained.

We found that the major rice-growing plains in the west and north of Antananarivo had no apparent effect on urban malaria transmission. Improvements in rice field irrigation systems were not investigated, but may have contributed to the absence of *An. funestus. *Muturi *et al*. (2008) found that the irrigation of rice crops decreased the risk of malaria transmission by *An. funestus *but had no effect on malaria transmission by *An. arabiensis *[30]. In 2003, Cot *et al*. (2006) demonstrated the presence of *An. arabiensis *and *An. funestus *within the houses of malaria patients living close to flooded rice fields [18]. Entomological surveys conducted from 2003 to 2005 in Antananarivo city identified only *An. arabiensis *as a vector of malaria [31]. Many authors have conducted study about environmental factors or urban malaria risks. Jacob *et al. *found that the proportion of aquatic habitats containing breeding sites for anopheline mosquitoes was higher at land use - land cover sites that had changed than at lad use - land cover sites that had not changed, in the urban area of Kisumu. They suggested that change could influence the distribution of habitats for anopheline larvae [32].

Jambou *et al*. (1998) observed an overall parasite index of 2.6% for health care centres after the epidemic. Almost all cases confirmed by parasitological examination concerned patients who had stayed in an area of hyperendemic malaria or had been in contact with an individual who had been to an area of high malaria transmission [33]. Cot *et al*. (2006) found only 1.9% positive cases in February 2003 and 1.5% positive cases in July 2003, [18]. Another five confirmed malaria cases were identified in a proximity survey of these cases, none with any history of travel [18]. That led us including the health centre close to this zone even it is not a public health centre. Urban malaria cross surveys in some African countries have reported similar results. In Dar es Salam (Tanzania) 5.2% of presumed cases were confirmed positive at health care centres. A lower frequency of positive cases was observed in Cotonou (Benin), where 1.8% of the 386 fever cases were positive for malaria. Our findings confirmed low malaria incidence and the persistence of a malaria transmission pocket. It showed similarity with surveys cited above. According to Baudon *et al*. (1996), urban malaria in the future may be characterised by an overall decrease in incidence but an increase in the severity of clinical symptoms, due to an absence of immunity [34].

Randremanana *et al*. (2001) showed that 1.9% of the city's population lives in zones at risk of flooding and that 26% of the population lives in zones in which 75% of the habitable surface is liable to flooding [29]. Cohen *et al*. (2008) reported a strong correlation between altitude and predicted wetness and showed that both variables were associated with malaria in households [35]. Our study showed geographical and environmental suitability for mosquito vector and increasing of malaria cases during the wet season.

This lack of correlation may result from a lack of statistical power, due to the low incidence of confirmed malaria cases. This study was therefore limited to the development of a descriptive model of the presence of breeding sites, vectors and the localisation of confirmed malaria cases. In future studies, it may be possible to assess the risk of urban malaria in Antananarivo by an extensive study of imported malaria cases, coupled with surveillance of the vector population, focusing particularly on the resurgence of *An. funestus*.

## Conclusion

Our findings confirm that malaria constitutes no real threat to the urban areas of Antananarivo. Of the presumed cases, 5.1% were confirmed with Polymerase Chain Reaction. Travel report was considered as related to the occurrence of malaria cases. *An. arabiensis *remained as the potential vector of the urban area of Antananarivo, rice fields are their principal breeding sites. Since the last epidemic of malaria in the highlands of Madagascar, the incidence of urban malaria in Antananarivo remained very low, although a pocket of transmission has persisted. Geographical and environmental factors did not show direct relationship with malaria incidence but they seem ensuring suitability of vector development. Absence of relationship may be due to a lack of statistical power. Despite the presence of *An. arabiensis*, scarce parasitic reservoir (mosquitoes not infected) and rapid access to health care do not constitute optimal conditions to a threatening malaria transmission. Imported malaria case is suggestive to sustain the pocket transmission in Antananarivo.

## Abbreviations

PCR: Polymerase Chain Reaction; ETM+: Enhanced Thematic Mapper; USAID: United State Agency for International Development; ASA_IMP_1P: Advanced Synthetic Aperture Image Mode Precision; DEM: Digital Elevation Model; VV: Vertical transmission Vertical reception; DNA: Deoxyribonucleic Acid; ELISA: Enzyme-Linked ImmunoSorbent Assay.

## Competing interests

The authors declare that they have no competing interests.

## Authors' contributions

JR, RR carried out the entomological study, fieldwork and analysis of results. FR, LR carried out the epidemiological study. RR performed the Polymerase Chain Reaction amplification tests on blood samples. VR did the risk analysis. JPR, FR, RRV performed the spatial interpretation of remotely sensed data. FR drafted the manuscript. All the authors read and approved the final manuscript.

## Pre-publication history

The pre-publication history for this paper can be accessed here:

http://www.biomedcentral.com/1471-2334/10/173/prepub
